# Association between Boarding of Frail Individuals in the Emergency Department and Mortality: A Systematic Review

**DOI:** 10.3390/jcm13051269

**Published:** 2024-02-23

**Authors:** Pasquale Iozzo, Noemi Spina, Giovanna Cannizzaro, Valentina Gambino, Agostina Patinella, Stefano Bambi, Ercole Vellone, Rosaria Alvaro, Roberto Latina

**Affiliations:** 1 Department of Biomedicine and Prevention, University of Rome “Tor Vergata”, Via Montpellier, 1, 00133 Rome, Italy; ercole.vellone@uniroma2.it (E.V.); rosaria.alvaro@uniroma2.it (R.A.); 2 Anesthesia and Intensive Care Unit, Emergency Department, Azienda Ospedaliera Universitaria Policlinico “Paolo Giaccone”, Via del Vespro, 129, 90127 Palermo, Italy; noemi.spina@policlinico.pa.it (N.S.); giovanna.cannizzaro@policlinico.pa.it (G.C.); valentina.gambino@policlinico.pa.it (V.G.); agostina.patinella@policlinico.pa.it (A.P.); 3 Department of Health Sciences, University of Florence, Viale GB Morgagni, 48, 50134 Florence, Italy; stefano.bambi@unifi.it; 4 Department of Nursing and Obstetrics, Wroclaw Medical University, 51-618 Wrocław, Poland; 5 Department of Health Promotion Science, Maternal and Infant Care, Internal Medicine, and Medical Specialities (PROMISE), University of Palermo, Piazza delle Cliniche, 2, 90127 Palermo, Italy; roberto.latina@unipa.it

**Keywords:** emergency department, boarding, systematic review, length of stay

## Abstract

(1) **Background:** Older patients who attend emergency departments are frailer than younger patients and are at a high risk of adverse outcomes; (2) **Methods:** To conduct this systematic review, we adhered to the Preferred Reporting Items for Systematic Reviews and Meta-Analyses (PRISMA) Guidelines. We systematically searched literature from PubMed, Embase, OVID Medline^®^, Scopus, CINAHL via EBSCOHost, and the Cochrane Library up to May 2023, while for grey literature we used Google Scholar. No time restrictions were applied, and only articles published in English were included. Two independent reviewers assessed the eligibility of the studies and extracted relevant data from the articles that met our predefined inclusion criteria. The Critical Appraisal Skills Program (CASP) was used to assess the quality of the studies; (3) **Results:** Evidence indicates that prolonged boarding of frail individuals in crowded emergency departments (Eds) is associated with adverse outcomes, exacerbation of pre-existing conditions, and increased mortality risk; (4) **Conclusions:** Our results suggest that frail individuals are at risk of longer ED stays and higher mortality rates. However, the association between the mortality of frail patients and the amount of time a patient spends in exposure to the ED environment has not been fully explored. Further studies are needed to confirm this hypothesis.

## 1. Introduction

The aging of the world’s population is accelerating rapidly, from 900 million people aged 60 and over in 2015 to an estimated 2 billion people by 2025, which will have profound implications for the healthcare system [1]. As the population ages, there is a clear need for emergency services to meet the expected increase in demand for high-quality care for frail older adults [2]. According to García-Peña et al. [3], this is the primary reason for the large increase in health system costs. Increasing average age and age-related problems have led to questions regarding the concept of frailty. Although not an inevitable condition of aging, frailty is mainly manifested in older persons by a dynamic state of vulnerability with weakness and a reduction in physiological reserve which entails an increased risk of lower quality of life, falling, institutionalisation, disability, and death [4,5,6,7]. Frailty is prevalent in all countries and is a leading contributor to functional decline, increased risk of adverse outcomes, and early mortality in older adults [8]. Up to 60% of older adults who present to the ED are frail and have higher ED admission rates, longer lengths of stay, increased probability of admission, and higher mortality than non-frail patients [9,10]. Because the two main measures of time spent in the ED are length of stay (LOS) and boarding, we considered both to assess waiting time. García-Peña et al. [3] argued in their study that adults over 65 constitute almost half of all deaths in EDs, and length of stay appears to be an important factor associated with mortality. According to Liu et al. [6], although aging does not affect all older adults in the same way, frailty is the strongest predictor of adverse health outcomes, and early identification may improve outcomes for older patients who access Emergency Departments (EDs). Since there is no universal definition of frailty and many studies conducted in emergency departments have identified frail patients without measuring frailty, an established frailty assessment tool is needed. The international guidelines recommend frailty screening in emergency departments [4]. Therefore, a multidisciplinary team of trained healthcare professionals, especially those working in the ED, should have appropriate knowledge and tools to recognise and effectively manage this typology of patients to achieve a better clinical outcome [11]. In particular, there is still no appropriate tool for frailty screening in the emergency department [4,5]. It is important to identify the goals of care at the time of the older patients’ presentation to the ED, to improve the delivery of care, and to focus on the symptoms [6]. This systematic review aimed to summarise the evidence regarding the association between ED boarding and mortality in frail patients.

## 2. Materials and Methods

We designed a systematic review and it was registered in PROSPERO with the number CRD42023417069. The PICO format was used to define the research question, as recommended by the PRISMA guidelines [12]: where the population was frail patients waiting in the ED, the comparator was non-frail patients waiting in the ED, and the outcome was the mortality of frail patients in relation to ED boarding time. 

## 3. Methodological Quality and Risk of Bias

Two independent reviewers (G.C. and N.S.) critically appraised eligible studies using the Critical Appraisal Skills Program (CASP) [13]. The results of the critical appraisal of the included studies, according to the search criteria, are summarised in Table 1. The overall quality of the studies was high according to the CASP method. In fact, according to the CASP method, 12 questions must be answered: (Q1) Did the study address a clearly focused issue? (Q2) Was the cohort recruited in an acceptable way? (Q3) Was the exposure accurately measured to minimise bias? (Q4) Was the outcome accurately measured to minimise bias? (Q5) Have the authors identified all important confounding factors? Have they taken into account the confounding factors in the design and/or analysis? (Q6) Was the follow-up of subjects complete and long enough? (Q7) What are the results of this study? (Q8) How precise are the results? (Q9) Do you believe in the results? (Q10) Can the results be applied to the local population? (Q11) Do the results of this study fit with other available evidence? (Q12) What are the implications of this study for practice? A “yes”, “no”, or “cannot say” was recorded for each question. The scoring system that was used for critical appraisal of the results considered a paper methodologically high if it had a number of yellow points (cannot say) less than or equal to three. Otherwise, it was considered medium if the number of balls was equal to four and methodologically poor if the number of yellow points was greater than four. One red ball corresponds to two yellow balls. Most observational studies had a clear focus on the issue, and the cohort was reconstructed acceptably, so the opportunity for selection bias was reduced. However, in almost all studies, the authors did not accurately measure exposure to minimise bias, which increases the risk of information bias arising from these studies. Confounding factors and strategies to address them were identified in eight of the thirteen studies (>50%).

## 4. Frailty Assessment

Frailty is often not analysed in the same way across studies, and according to Liu et al. [6], many studies that identified patients as frail did not always measure frailty using an instrument. In order to provide greater clarity on the instruments analysed, we have included them in Table 2. This table not only indicates the type of instrument associated with each scale, but also provides details of the scales’ characteristics, their validation status, and the reference articles. All studies in the review specified what was meant by frail patients (see Table 3), but not all did so in the same manner. Four studies used the Clinical Frailty Scale (CFS) to define frailty [6,7,16,17]. Two studies used Fried’s definition of frailty phenotype [2,9]. One study used the Hospital Frailty Risk Score (HFRS) criterion [11], whereas two studies used the FRAIL questionnaire [3,5]. Only one study used two instruments together: the CFS and Fried’s frailty phenotype instrument [14]. One study used the Comprehensive Geriatric Assessment (CGA) [10], whereas only one study used the Dutch Safety Management System (VMS) [4].

## 5. Boarding or ED Length of Stay and Outcomes

Within the reviewed studies, a number of outcomes were analysed, as shown in Table 3. Numerous studies have observed an association between longer boarding, higher frailty, and mortality as outcomes [2,3,6,10,11,14,15]. According to García-Peña and collaborators [3], many studies have investigated the negative effects of ED length of stay on the health and quality of life of older patients, and how length of stay in the emergency department was relevant for patients who were referred for hospitalisation. According to Cardona et al. [14], patients who died in the hospital had a significantly higher LOS than those who survived. Therefore, LOS deserves attention, and new studies should be conducted.

## 6. Inclusion Criteria

This review included randomised and controlled clinical trials and observational studies, including retrospective and prospective cohort studies. On the other hand, incomplete studies, articles with insufficient data, or those not available in the full text were excluded. No time restrictions were applied, and only articles published in English were included. No geographic limitations were included. The affected population was frail subjects (as defined by the authors) aged 60 years or older who were admitted to the ED as a patient and boarded there, waiting for an inpatient bed. The commonly accepted description of frailty in the literature is a condition of latent vulnerability that leads to a substantially increased risk of falls, loss of autonomy, disability, and a higher risk of acute hospital admission and death [4,6,18,19,20]. Its assessment criteria are still the subject of debate within the scientific community in the emergency department because there is no universal definition of frailty, and many studies have been conducted in emergency departments to identify frail patients without measuring frailty [6]. Instead, boarding refers to holding patients in the ED or a dedicated area while waiting for an inpatient bed [21,22], whereas LOS indicates the total time in the emergency department from arrival to discharge. Even in the case of the definition of boarding, as pointed out in the review, many articles refer to boarding or length of stay (LOS) without providing a precise definition. For this reason, we consider both.

## 7. Information Source, Search Strategy, and Study Selection

**Figure 1:**
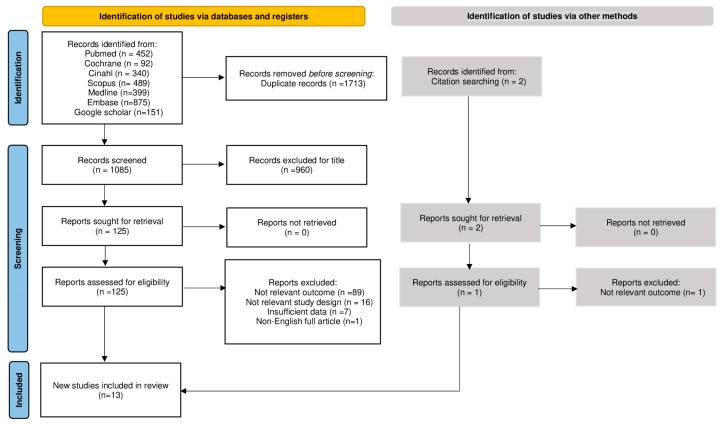
PRISMA flow diagram.

In this review, only English-language articles were included, and the search was conducted until May 2023 without a time filter. Seven databases were screened: PubMed, Embase, OVID Medline^®^, Scopus, CINAHL via EBSCOHost, and Cochrane Library. Google Scholar was used for gray literature. The research in every database was achieved using the same keywords and by following the syntax rules of each database. The complete search strategy is provided in Appendix A. The article selection process was performed according to the PRISMA Statement. All citations were uploaded to Mendeley Desktop and the Rayyan web application [23] to facilitate the independent work of the reviewers, and duplicates were removed. Title and abstract screening was performed by two independent reviewers (V.G./A.P.). All the studies were screened according to the inclusion and exclusion criteria, and articles that did not meet the inclusion criteria were excluded. Of each study that was considered relevant based on title or abstract, the full text was evaluated by two independent reviewers (N.S., G.C.) who assessed its eligibility. Full-text studies that did not satisfy the inclusion criteria were not included, and the rationale for exclusion was reported in the PRISMA flow diagram (Figure 1). Any disagreements between the reviewers were resolved through careful communication. The authors of these studies were not contacted for further information.

## 8. Outcomes, Data Extraction, and Analysis

An electronic data extraction form for the systematic review, previously prepared and agreed upon by the authors, was used for data extraction. Two reviewers (N.S., V.G) who worked independently obtained the following information from each inclusion study: author’s names, year of publication, country, design, sample and setting, number of participants, outcomes (including mortality, total LOS, and boarding), and definition of frailty and length of stay (Table 3).

## 9. Results

The search identified 2798 records; 1713 of these were duplicated, and 13 studies were included in this systematic review. All 13 studies were published. We retrieved the following full texts: two retrospective cohort studies, five prospective cohort studies, one retrospective case-control study, two prospective observational studies, one cohort single-site study, one prospective before-and-after study, and a secondary analysis of a prospective cohort study. Studies were conducted in a wide range of countries, including the Netherlands (n = 1), Brazil (n = 1), China (n = 1), Taiwan (n = 1), Mexico (n = 1), Ireland (n = 1), Switzerland (n = 2), Germany (n = 1), France (n = 1), the United Kingdom (n = 2), and Australia (n = 1). The PRISMA flow diagram [24] in Figure 1 shows the results of the search, as well as the process of inclusion of studies and their selection. Our systematic review aimed to analyse the association between boarding times in emergency departments and mortality in frail patients aged 60 years and older. Upon analysing the included studies, it becomes evident that there is a pressing need to design an alternative pathway for assessing frail and older patients. In a prospective cohort study carried out by van Dam et al. [4], in which 889 patients aged ≥70 years were evaluated, patients’ functional decline, institutionalisation, and mortality were assessed. According to van Dam, older patients stay longer in the ED and have a high risk of adverse outcomes. The study shows that at each follow-up, mortality increased from 4% (38) in the first month to 9% (76) in the second month and 14% (107) in the sixth month. Indeed, the study confirmed the increased risk of adverse events in a substantial portion of older patients visiting the emergency department, supporting the need for frailty screening in the ED. The study performed by Aprahamian et al. [5], on the other hand, included a total of 316 older patients aged 60 years and older. Of these, 25.6% were defined as frail according to the FRAIL questionnaire and the average length of hospital stay was 5.43 ± 5.6 days. Of these patients, 52 died and 55 returned to the emergency department, and frailty was associated with an odds ratio of 2.18 for death at 6 months (95% CI = 1.10–4.31; *p* = 0.024). The author argues that the assessment of frailty is important to identify a patient at increased risk of death and that further studies, especially randomised trials, are needed to investigate such an important condition in the emergency department. Liu and associates [6] also believe that if frailty were identified earlier in the ED, the risk of increased length of stay (LOS), adverse events, readmissions, and mortality would be reduced. In their study, 350 adults aged 60 years and older were recruited, 65.7% of whom were older than 75 years. The median length of hospital stay was 12 (7, 17) days. The FRAIL scale was used to identify frail patients, with a score of ≥3 defined as frail. Using this scale, 156 participants were classified as frail, with a prevalence of 44.6%. The 28-day mortality of this group compared to the non-frail group was 16.7% vs. 3.6%, while the LOS was 13% (8.25, 21.75) in the frail group compared to 10% (6, 15) in the non-frail group (all *p* < 0.01). This shows the association between the FRAIL scale and higher 28-day mortality, as well as LOS and hospital readmission, and the importance of the accurate identification of frailty to predict negative outcomes in the ED [6]. Lin et al. [9] used a before-and-after design aimed at evaluating Comprehensive Geriatric Assessment (CGA) screening in older patients in the ED (aged 75 years and older) and their clinical outcomes. They found a high prevalence of geriatric syndromes in older adults visiting the ED, where 76.5% of the patients had frailty. It has been observed that up to 60% of the older people who visit the ED are frail and have higher ED utilization rates, longer lengths of stay, increased probability of admission, and higher mortality rates. The clinical outcomes showed that older patients with frailty had an increased risk of mortality at three months. It also showed that with the implementation of CGA in the ED, three-month mortality, ED readmissions, and hospitalizations were significantly reduced [9]. According to García-Peña et al. [3], an aging population poses major challenges to societies and healthcare systems worldwide. Healthcare systems do not respond effectively or efficiently, particularly because they are designed to treat the injuries or acute medical conditions of a younger population, placing frail older adults on the back burner. In their study, they evaluated 1406 adults aged 60 years and demonstrated a high mortality rate among the older adults (21.7%) in two non-specialised Mexican public hospitals. They showed that 71% of all deaths occurred during hospitalization after an ED visit and referral, and that the factor associated with mortality that appears to be important is the length of stay in the ED. In all analyses, this variable was found to be significant and could be attributed to the presence of inappropriate care in the ED. In fact, factors related to the healthcare process were statistically significant for delayed ED arrival (*p* = 0.048), number of hours in the ED [111.62 (±SD 63.18) for non-survivors vs. 97.84 (±SD 68) for survivors] (*p* < 0.001), and any referrals to hospitalisation (*p* ≤ 0.001). According to García-Peña, the combination of an aging population and fragmented health and social systems is an inadequate response to this new scenario. In this setting, the results of this study showed that mortality is high in patients over 60 years of age who present to the ED, and that the factors associated with mortality include the organisation of health care and the length of stay in the ED, which has a negative impact on the functionality and quality of life of older patients. However, Gaffney et al. [10] took a different approach in their secondary analysis of a prospective study of 191 patients aged ≥70 years attending the emergency department of a university hospital. They introduced a new tool called the Surprise Question (SQ) which is a brief assessment that is considered useful in predicting mortality. Initially, SQ was used to identify patients suitable for palliative care services, but it has also been studied in emergency departments (EDs) and has demonstrated short-term (one month) and long-term (one year) predictive validity for death among patients admitted to the ED. In this study, SQ was scored by ED physicians and was asked of those who met the inclusion criteria after a detailed CGA to determine their frailty status. The results of the study showed that all 56/191 (29%) patients screened were SQ-positive and that SQ-positive patients were frailer, had a longer LOS (68% vs. 44%, *p* = 0.008), and had higher mortality (36% vs. 11%, *p* < 0.001). Another crucial point was highlighted by Rueegg et al. [16] in their prospective cohort study of the ED of a care centre in Switzerland. In the definitive study cohort of 2191 patients, 1-year all-cause mortality was 17% (n = 372), and higher levels of frailty were associated with higher hazard ratios. In a retrospective study by Rauch et al. [11], including 13,451 elderly patients aged ≥75 years who presented to the emergency room, comparisons between frail and non-frail patients were made. Of these, 44.8% (n = 6025) were considered frail. They found significant differences between the two groups, specifically higher ED arrival rates for frail patients and longer ED stays of about half an hour (*p* < 0.001, 95% CI 27.9–30.6 LOS difference in min). These results suggest that intervention teams trained to deal with frail patients are needed in EDs, especially during the day, to reduce the high demand for ED care. Maarek et al. [15] showed in their prospective study of 298 patients with non-severe comorbidities (aged 75 years or older) who presented to the ED that the risk of 30-day all-cause mortality was 10 times higher for frail patients than for non-frail patients. The prevalence of frailty in the included population was high (52%); specifically, the rate of 30-day mortality was significantly higher in the frail group than in the non-frail group, 11% versus 1% (*p* = 0.0002), and the median length of stay was significantly higher in the frail group than in the non-frail group, 5 (0–12) days versus 0 (0–3) days (*p* < 0.0001). The 30-day readmission rate was also significantly higher in the frailty group than in the non-frailty group (18% vs. 10%, respectively; *p* = 0.04). In conclusion, frailty should be evaluated in older patients, including non-comorbid patients presenting to the emergency department, and intervention should be initiated. In the analysis by Lewis et al. [2], 468 patients aged 75 years and older were recruited. Of these, 356 (76.1%) had sufficient data to be classified as frail using the Frailsafe classification. A positive Frailsafe screen was predictive of death within 180 days of emergency department presentation and remained so after adjustment (AOR = 3.23, 95% CI 1.45–7.19, *p* = 0.004). A positive Frailsafe screen was also a predictor of length of stay >28 days (AOR 3.42, 95% CI 1.41–8.31, *p* = 0.007). The study showed that older patients who were admitted to the hospital were at a high risk of negative outcomes, with increased length of stay, higher rates of functional and cognitive decline, increased risk of readmission and hospitalisation after discharge, and mortality. Kabell Nissen and collaborators [17] show that combining information from aggregated vital signs and frailty levels measured by the CFS in the ED facilitates early and accurate recognition of patients over 65 years of age at risk of 30-day mortality. This suggests that a simple judgment-based evaluation of frailty provides clinical information that has an important clinical correlation with the severity of acute illness, and may provide better support for clinical decision making in the ED in older patients, emphasising the need for impact studies that assess the impact on clinical outcomes other than classic service-related outcomes (mortality, readmissions, and length of stay). Elliott et al. [7] studied care and outcomes for older persons (65 years old) registered after presentation in the ED. Data were obtained from 52,562 individuals during the study period. This represents a very large sample size under study on the Clinical Frailty Scale applied to ED triage to identify the risk of adverse outcomes in older adults. Total mortality was 24% at 2 years, but increased with Clinical Frailty Scale categories; in fact, the Clinical Frailty Scale was strongly related to the risk of an increase in hospital admissions and death. Increasing Clinical Frailty Scale scores generally increased the cumulative number of hospital days after the index ED visit (>30 or 180 days). Cardona et al. [14] demonstrated that the Clinical Prediction Tool Criteria for Screening and Triaging to Appropriate Alternative Care (CRISTAL), based on existing objective parameters has a good discriminative ability to identify older patients at risk of death. They confirmed that the mortality at the end of follow-up for all participants was 10.1% (116) in Australia and 13.6% (184) in Denmark. The mean follow-up period was 124 days in Australia (IQR 105–170) and 97 days in Denmark (IQR 92–149), and most deaths occurred in the first 4 months. Those who died in the hospital had a significantly longer length of stay than those who survived in both health systems.

## 10. Discussion

This review examined the association between boarding and patient outcomes, including mortality and adverse events. Many of the articles analysed, including those later included in the review, do not always clearly define the concept of frailty or patient boarding in the ED because there is no single definition around the world [6]. This creates difficulties in assessing and measuring the phenomenon, as it is not always easily quantifiable, particularly when measured differently from one study to another. This supports our decision to consider valid exposure, whether it is defined as boarding or LOS. The problems of boarding and EDLOS in ED are multifactorial and complex phenomena. These causes can be classified into three categories: input, throughput, and output factors. These different factors are independent of each other, but are closely related and influenced by other factors. Identifying and understanding these three factors is a useful guideline both in hospital settings and in the healthcare system in general [25]. The Input Factors include waiting time, number, severity, and complexity of patients arriving at the ED. Throughput Factors represent the hospital’s internal factors; thus, the time between patient admission and the medical decision regarding the patient after diagnosis follows the decision: discharge, admission, and transfer. These factors are operator-dependent. Output factors include patient boarding in the ED, availability of hospital beds, and transport delays (both internal and external) in leaving the ED. A lack of hospital beds appears to be a major cause of overcrowding [26]. Together, these factors increase EDLOS and the possibility of adverse outcomes. This systematic review included studies conducted in eleven different countries. This means that a wide range of hospital settings and emergency department models were considered, which contributed to the diversity of settings and systems within the included studies. Each country has its own model of care, emergency departments, population, and socio-demographic factors that may contribute to differentiating study results. Despite the heterogeneity of the studies (countries, emergency department models, frailty status of patients 60 years of age or older), the LOS variable was found to be statistically significant for frail patients compared with non-frail patients. Two studies documented an association between the length of stay in the ED and mortality [3,14]. This may be attributed to the presence of insufficient care processes in the ED; however, the main problem is that not all studies evaluating the association with mortality have focused on this. Eleven studies analysed the association between frailty and higher mortality [2,4,5,6,7,9,10,14,15,16,17]. Indeed, most studies support the use of frailty to better predict adverse outcomes, although there is still no gold standard for measuring frailty [5]. Therefore, focused intervention in these patients by an appropriately trained team of experts could lead to improved clinical outcomes, as highlighted by Liu and collaborators [6]. Five studies documented an association between frailty and length of time spent in the ED. Frailty is associated with longer time spent in the ED [2,6,10,11,15]. This may be because frail patients are older and have more comorbidities, and their management is more complex and requires more time. Moreover, eight studies documented an association between frailty and institutionalisation [2,3,4,6,7,9,10,15]. Four studies reported an association between age and mortality rate. Older age is associated with higher mortality [3,4,7,14]. This is because older adults require more ED resources and have more complicated healthcare needs than other age groups do. In fact, these are the primary reasons for the large increase in health system costs [3]. According to García-Peña et al. [3], this is because the current emergency department model is designed to manage acute care and injured patients, not geriatric patients with multimorbidities [3]. The inadequate response to this new scenario is the combination of an aging population and fragmentation of health and social systems [3]. 

## 11. Limitations

The studies included in this systematic review were published only in English. Many studies have included mortality as the primary outcome after eliminating confounding factors. However, this same level of analysis was not used for secondary outcomes such as LOS and boarding. Meta-analysis was not performed because of the heterogeneity of mortality-related time frames. This limits the ability to derive a statistical interpretation of the results. Another important limitation is that the concept of frailty has not been described equally by the authors; therefore, there is no single definition.

## 12. Conclusions

Our systematic review focuses on frail patients who may be at risk of a longer ED stay and may also incur a higher mortality rate. Other literature reviews have analysed the increased mortality rate related to boarding but not to frail patients. In contrast, the “frail patient-boarding-mortality” correlation was investigated by adding new data to the literature; therefore, our systematic review provides useful evidence and lays the foundation for further studies. In fact, in the articles analysed, it seems that the concept of frailty or boarding is not expressed in the same way by all authors, which limits the measurement of the phenomenon and does not allow quantitative data analysis and reliable conclusions. Based on our review, it is necessary to design randomised controlled trials to investigate significant and prevalent geriatric conditions, such as frailty, in acute care settings, and their association with prolonged boarding and mortality, to develop targeted interventions to better protect frail patients who visit the ED.

## Figures and Tables

**Table 1:** Critical appraisal skills program (CASP) results of the included observational studies.

	Study ID	Q1	Q2	Q3	Q4	Q5	Q6	Q7	Q8	Q9	Q10	Q11	Q12	Overall
1	Cardona et al., 2018 [14]	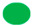	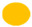	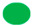	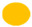	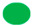	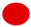	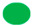	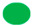	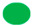	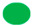	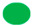	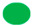	Medium
2	García-Pena et al., 2018 [3]	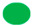	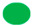	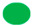	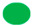	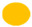	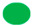	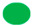	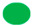	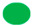	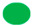	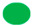	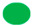	High
3	Aprahamian et al., 2019 [5]	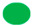	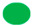	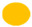	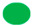	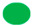	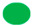	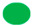	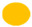	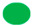	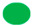	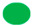	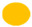	High
4	Rauch et al. 2019 [11]	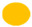	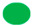	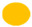	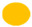	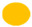	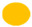	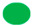	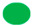	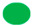	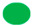	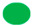	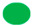	Low
5	Elliot et al., 2021 [7]	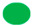	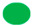	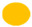	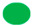	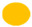	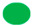	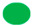	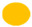	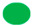	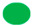	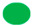	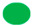	High
6	Lewis et al., 2020 [2]	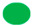	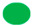	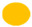	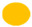	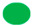	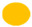	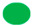	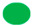	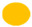	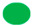	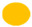	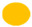	Low
7	Liu et al., 2020 [6]	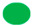	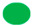	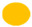	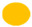	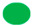	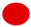	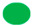	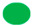	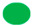	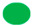	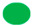	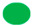	Medium
8	Maarek et al., 2020 [15]	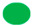	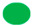	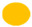	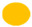	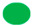	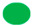	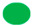	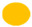	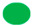	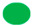	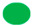	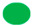	High
9	Lin et al., 2021 [9]	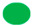	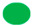	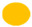	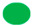	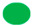	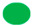	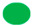	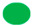	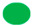	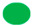	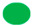	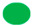	High
10	Rueegg et al., 2021 [16]	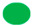	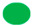	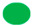	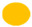	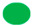	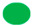	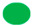	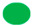	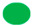	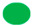	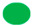	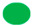	High
11	Van Dam et al., 2021 [4]	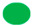	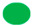	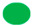	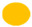	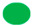	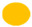	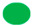	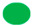	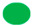	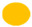	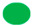	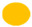	Medium
12	Gaffney et al., 2022 [10]	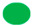	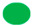	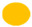	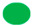	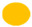	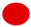	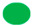	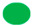	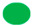	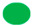	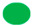	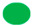	Medium
13	Nissen et al., 2022 [17]	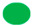	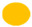	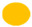	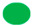	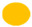	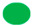	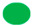	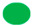	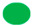	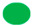	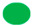	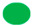	High
		Legend: 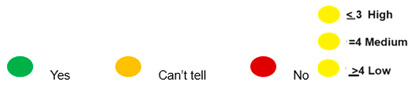												

**Table 2:** Frailty assessment tool.

Scale	Instrument Type	Characteristics	Validation
Clinical Frailty Scale (CFS) [6,7,14,16,17]	Frailty screening instrument	The Clinical Frailty Scale is a 9-point scale, delineating distinct levels of frailty. Beginning at 1 (representing very fit), the scale progresses through increasing stages of frailty, reaching 8 (indicating very severely frail), and concluding at 9 (signifying a terminally ill condition).	Criterion validity
Fried phenotype of frailty [2,9,14]	Frailty screening instrument	The five frailty criteria are weight loss, exhaustion, low physical activity, slowness, and weakness. The cumulative score of these criteria categorises individuals into one of three frailty stages (or groups): not frail (score 0), pre-frail (score 1–2), and frail (score 3–5).	Criterion validity
Hospital Frailty Risk Score (HFRS) [11]	Frailty risk assessment instrument	The Hospital Frailty Risk Score (HFRS) is employed to detect frailty in hospitalised patients, assigning a numerical score based on the presence of specific ICD-10 codes documented in the individual’s previous hospitalization records. The risk of frailty is categorised as low (<5 points), intermediate (5–15 points), or high (15 points). A patient with at least 5 points is considered frail.	Criterion validity
Frail Scale [3,5]	Frailty screening instrument	The Frail Scale is composed of 5 items: fatigue, resistance, ambulation, illness, and loss of weight. Scores ranged from zero to five points (1 point for each component; 0 = best to 5 = worst) and represent frail (3–5), pre-frail (1–2), and robust (0) health status.	Internal consistency (Cronbach’s Alpha value)
Comprehensive Geriatric Assessment (CGA) [10]	Frailty assessment instrument	Within the Comprehensive Geriatric Assessment (CGA), evaluation tools encompass two frailty measures: the FRAIL scale and the Groningen Frailty Indicator (GFI). The FRAIL scale is a concise questionnaire consisting of five items, with scores ranging from zero (not frail) to five (most frail). Individuals scoring one are categorised as pre-frail, while a score of two or more indicates frailty. The GFI assesses frailty across physical, cognitive, social, and psychological domains, utilizing a cut-off threshold of ≥4 out of 15.	Internal consistency (Cronbach’s Alpha value)
Dutch Safety Management System (VMS) [4]	Frailty risk assessment instrument	The VMS screener identifies patients (aged 70 years or older) at risk for delirium, falls, malnutrition, and functional impairment who require preventive measures. The VMS score was calculated by adding up all positive domains, resulting in a score ranging from zero to four. For patients aged 70 to 80 years, a score of ≥3 indicates frailty; in patients aged ≥80 years, a score of 1 indicates frailty.	Internal consistency (Cronbach’s Alpha value)

**Table 3:** Summary of included studies.

Author (Year), Country	Design	Sample and Setting	Outcomes	Quality of Evidence	Frailty Definition	ED Length of Stay (LOS) or Boarding Definition	Comments
García-Peña et al. (2018) [3], Mexico	Retrospective cohort study	≥60 years, ED N = 1406	Mortality at 120 days (21.7%), the length of stay (in hours) in the ED (4.8 h).	High	YES	YES	This study analyses all causes of death, and one factor associated with mortality that appears to be important is the length of stay in the ED. This variable was statistically significant in all analyses.
Cardona et al. (2018) [14], Australia	Prospective cohort study	≥65 years, ED N = 2493	90-days mortality (10.1% in Australia and 13.6% in Denmark) and ability of CriSTAL to predict in-hospital death.	Medium	YES	NR	This study analyses the deaths at 90 days in two cohorts (Australia and Denmark) and specifies that those who died in the hospital had a significantly longer mean length of stay than their counterpart who survived in both healthcare setting.
Aprahamian et al. (2019) [5], Brazil	Prospective cohort study	≥60 years, ED N = 316	Death at 6 months (16.5%), readmission to ED (17.4%).	High	YES	NR	Frailty was related to an odds ratio of 2.18 for mortality at 6 months (95% CI = 1.10–4.31; *p* = 0.024), even after adjusting for age and sex. This suggests that frailty may be a predictive factor for death.
Rauch et al. (2019) [11], Germany	Retrospective case-control study	≥75 years, ED N = 13,451	Examines differences in arrival rates for frail vs. non-frail patients in detail and comparision of case complexity (ED length of stay).	Low	YES	YES	Comparing frail and non-frail groups, they found significantly higher levels for all examined variables (including ED LOS) in frail patients.
Maarek et al. (2020) [15], France	Prospective observational study	≥75 years, ED N = 298	30-day all-cause mortality (6%), length of stay, and emergency readmission within 30 days of initial discharge.	High	YES	NR	Their study showed that the frail patient group, compared with the non-frail patient group, had a higher risk of death, hospital readmission, and LOS.
Liu et al. (2020) [6], China	Prospective cohort study	≥60 years, ED N = 350	All-cause 28-day mortality (9.4%), ADL Dependency, mechanical ventilation (9.7%), LOS in hospital, and ICU readmission 30 and 90 days after discharge (14.6% and 24%).	Medium	YES	NR	Their study showed that the frail patient group, compared with the non-frail patient group, had a higher risk of death, hospital readmission, and LOS.
Lewis et al. (2020) [2], United Kingdom	Cohort single-site study	≥75 years, ED N = 468	Mortality at 180 days, length of stay of >14 and > 28 days, inpatient mortality, and reattendance within 30, 120, and 180 days of discharge after admission.	Low	YES	NR	A positive Frailsafe predicts an increased risk of mortality, hospitalization, and a combined 180-day outcome of mortality and hospitalization.
Van Dam et al. (2021) [4], Netherlands	Prospective cohort study	≥70 years, ED N = 889	Functional decline (20%), institutionalization (11%), and mortality (10%).	Medium	YES	NR	This study confirms that older patients in ED are at higher risk of adverse outcomes and they considered prolonged length of stay as an outcome measure.
Rueegg et al. (2021) [16], Switzerland	Prospective single-centre observational Cohort study	≥65 years, ED N = 2191	1-year all-cause mortality (17%).	High	YES	NR	This study shows that higher frailty levels were associated with higher mortality.
Lin et al. (2021) [9], Taiwan	Prospective Before-and-after study	≥75 years, ED N = 358	Revisits to the ED (30.7%), admission for hospitalization (20.4%) and mortality within three months (5.6%).	High	YES	NR	Outcomes revealed that older patients with frailty had increased three-month mortality, higher ED use, longer length of stay, increased probability of hospitalization, and higher mortality.
Elliot et al. (2021), [7] United Kingdom	Retrospective single-centre cohort study	≥65 years, ED N = 52,562	Length of stay, readmission, mortality (24% at two years) and associations of the Clinical Frailty Scale at ED with negative outcomes.	High	YES	YES	They used the Clinical Frailty Scale at ED triage to assess the risk of adverse outcomes in older patients. Results showed that the Clinical Frailty Scale applied at a single point was strongly associated with the risk of increased hospital use and death, even after adjustment for prognostically important covariates. Cumulative postoperative days in the ED (>30 or 180 days) were associated with increasing Clinical Frailty Scale scores.
Kabell Nissen et al. (2022) [17], Switzerland	Prospective single-centre observational Cohort study	≥65 years, ED N = 2250	30-day mortality (5.4%).	High	YES	NR	This study shows that a simple assessment of frailty can give clinical information that has high clinical interaction with acute disease severity and may provide better support for clinical judgment in the ED regarding older patients.
Gaffney et al. (2022) [10], Ireland	Secondary analysis of a prospective cohort study	≥70 years, ED N = 191	Length of stay (LOS, 8 days), frailty deter- mined by CGA and one-year mortality (18%).	Medium	YES	NR	Study showed that a large number of older people presenting to the ED are SQ-test-positive and this is associated with frailty, hospital admission, prolonged length of stay, and death within a year.

Legend: NR = not reported, N = number of patients presenting at Emergency Department (ED), LOS = Length of stay, CGA = Comprehensive geriatric assessment, SQ = Surprise Question, ICU = Intensive Care Unit.

**Figure A1:**
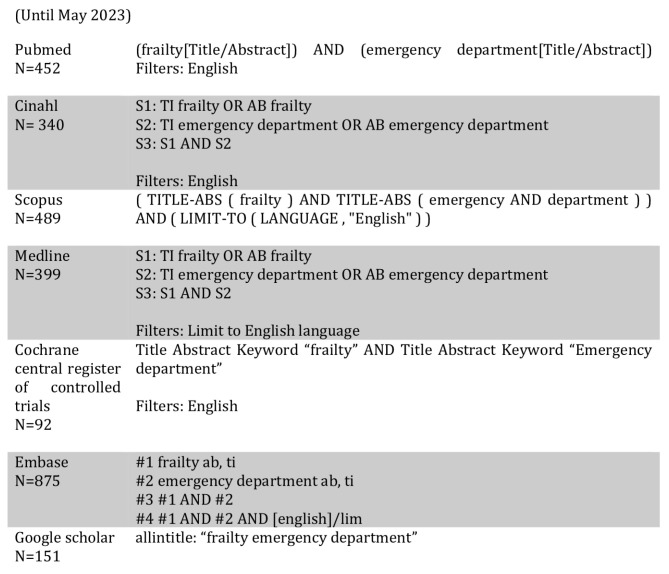
Search strategy.

## Data Availability

Not applicable.
